# Treatment of Refractory Bipolar Depression With Stereotactic Radiosurgery Targeting the Subgenual Cingulate Cortex

**DOI:** 10.7759/cureus.57904

**Published:** 2024-04-09

**Authors:** Hugh B Solvason, Neelan J Marianayagam, Scott G Soltys, Alan F. Schatzberg, Charles DeBattista, Terence Ketter, Po Wang, Steven D. Chang, David Spiegel, John R Adler

**Affiliations:** 1 Department of Psychiatry, Stanford University School of Medicine, Stanford, USA; 2 Department of Neurosurgery, Stanford University School of Medicine, Stanford, USA; 3 Department of Radiation Oncology, Stanford University School of Medicine, Stanford, USA

**Keywords:** bipolar depression, neuromodulation, radiosurgery, subcallosal cortex, treatment resistance

## Abstract

Background

The subgenual cingulate cortex (SGC) has been identified as a key structure within multiple neural circuits whose dysfunction is implicated in the neurobiology of depression. Deep brain stimulation in the SGC is thought to reduce and normalize local metabolism, causing normalization of circuit behavior and an improvement in depressive symptoms. We hypothesized that nonablative stereotactic radiosurgery (SRS) to the SGC would reduce local metabolism and reduce the severity of depression in patients with treatment-resistant bipolar depression.

Methods

Under the FDA’s Humanitarian Device Exemption program, patients were screened for inclusion and exclusion criteria. Three volunteers meeting the criteria provided informed consent. Bilateral SGC targets were irradiated to a maximum dose of 75 Gy in one fraction. Subjects were followed for one year following the procedure with mood assessments (Hamilton Depression Rating Scale (HDRS), Clinical Global Impression-Improvement, Clinical Global Impression-Severity, and Young Mania Rating Scale), neurocognitive testing (Delis-Kaplan Executive Function System, Wechsler Adult Intelligence Scale III digit span, and California Verbal Learning Test II), and imaging. Further imaging was completed approximately two years after the procedure. Clinical improvement was defined as a ≥50% reduction in HDRS.

Results

Two of the three subjects showed clinical improvement in depressive symptoms during the follow-up period, while one subject showed no change in symptom severity. One of three subjects was hospitalized for the emergence of an episode of psychotic mania after discontinuing antipsychotic medications against medical advice but promptly recovered with the reinstitution of an antipsychotic. Sequential assessments did not reveal impairment in any cognitive domain assessed. For one of the three subjects, MRI imaging showed evidence of edema at 12 months post-SRS, which resolved at 22 months post-procedure. In a second of three patients, there was evidence of local edema at the target site at long-term follow-up. All imaging changes were asymptomatic.

Conclusion

Radiosurgical targeting of the SGC may be a noninvasive strategy for the reduction of severe depression in treatment-resistant bipolar disorder. Two out of three patients showed clinical improvement. While these results are promising, further study, including improvements in target selection and dosing considerations, is needed.

## Introduction

Bipolar disorder has a progressive course broadly characterized by an early onset of illness, highly recurrent and chronic major depressive episodes (MDEs), and increasing disability. Bipolar 1 disorder is associated with euphoric manias early in the course, while over time these euphoric states are replaced by dysphoric or mixed manic states, rapid cycling, and severe, chronic MDEs that are difficult to treat [1-9]. Even with the appropriate use of mood stabilizers and adjunctive treatments, sustained remission occurs in a minority of patients.

Treatment-resistant bipolar depression (TRBD) has a notably higher risk of morbidity and mortality than unipolar depression [10-12]. While the manic phase defines the illness, over the course of time, patients are most symptomatic in the depressed phase, in which the risk of suicide is greatest [2]. The high prevalence of all bipolar disorders [13], the increased chronicity and disability primarily associated with depressive episodes over the illness course [14], and the large proportion of patients with treatment-resistant bipolar disorder make the development of new strategies and technologies for TRBD a high priority.

The brain circuits associated with bipolar disorder include the subgenual cingulate cortex (SGC). The SGC has been postulated to be an important nexus in a web of connected prefrontal, subcortical, temporal, and limbic structures in which dysregulation is thought to be a key feature of the pathophysiology of mood disorders [15,16]. Volumetric MRI studies have shown marked atrophy of SGC in bipolar disorder [15,17], which may be reversible [18]. Volumetric cell counts and cytoarchitectural studies suggest the volume loss in SGC is primarily due to the loss of glial cells rather than neurons [19]. The SGC has been reported to be hypermetabolic in the depressed state, which then normalizes in those who respond to antidepressant treatment [20], although the relationship between metabolism in the SGC and treatment is complex [21]. Mood disorders have been postulated to be disorders of circuit hyperconnectivity [22], a finding consistent with the presence of a strong bidirectional structural neuroanatomic pathway between the SGC and many limbic and prefrontal structures [23]. It has been reported that depression is associated with greater connectivity of the SGC to the default mode network [24]. Another study showed that over the course of electroconvulsive therapy (ECT) treatment, there was a significant reversal of hyperconnectivity between the SGC and the bilateral hippocampus, bilateral temporal pole, and ventromedial prefrontal cortex [25]. An open study of deep brain stimulation (DBS) in the SGC has been published for 17 unipolar and bipolar subjects with treatment-resistant depression [26]. These results and others have been encouraging, with many patients experiencing persisting improvement or remission [27]. DBS is relatively safe; however, the implantation is an open neurosurgical procedure and carries associated risks such as infection, blood loss, and neurological disability.

Stereotactic radiosurgery (SRS) has been used extensively for the treatment of trigeminal neuralgia by targeting either the cisternal or dorsal root entry zone of the nerve, with a low incidence of facial sensory loss as a result of treatment [28-31]. This is one piece of evidence that suggests that, at moderate doses, radiation can exert nondestructive effects on neural function. Studies in animal models and in humans show that delivery of low-dose radiation of up to 80 Gy does not appear to result in cell death in the Gasserian ganglion, although there is local destruction of the myelin sheath and clear neuronal death in doses above 100 Gy [32-34].

We hypothesized that targeting SGC with focused radiation could safely reduce local metabolic activity and, similar to DBS, reduce depressive symptoms in TRBD. The dose of radiation used in this study was based on published clinical studies for the treatment of pain with trigeminal neuralgia [35].

## Materials and methods

This study was done as described in the FDA-approved Humanitarian Investigational Device Exemption (G090015/S010) titled “Radiosurgical Neuromodulation for Refractory Bipolar Depression.” Institutional IRB protocols were followed. Each subject was carefully screened and assessed, and a detailed discussion of the study and the potential risks and benefits ensued. This process was continued until the investigators were satisfied that the potential patient fully understood the study, the procedures, the potential risks, and the possible benefits of the radiosurgical procedure. The subjects were assessed by a psychiatrist, who was not otherwise involved in the study, to address the competence of the subject’s understanding of the risks and benefits of participation and to uncover special circumstances that might have created a context of undue influence on participation in the trial. Four subjects were consented to and screened for the study; one subject was excluded due to a preexisting structural abnormality on the subsequent screening MRI. The inclusion criteria for the study were as follows: primary diagnosis of bipolar depression per the Diagnostic and Statistical Manual of Mental Disorders, Fourth Edition, Text Revision (DSM IV-TR) criteria (study carried out between 2010 and 2012); duration of the current MDE of more than one year; Hamilton Depression Rating Scale-24 (HDRS-24) item greater than or equal to 20; treatment resistance: a history of failure to show clinical improvement after at least four different medication trials of adequate dose and duration; nonresponse or lack of sustained improvement after one course of ECT (six to 12 sessions); no viable treatment options at the time of enrollment; competent to understand the risks and potential benefits of the study; able to provide written informed consent for the full screening phase, as well as the treatment period of the protocol, including the baseline MRI, CT, and PET imaging; and signed consent form for participation in the study. The exclusion criteria were rapid cycling bipolar illness, history of schizophrenia, schizoaffective disorder, or psychosis; severe suicidal thoughts that may put the subject at risk of either attempted suicide or completed suicide for the duration of the trial, as determined by the investigator at the time of enrollment; current substance abuse or withdrawal from psychoactive substances, including alcohol, stimulants, or sedatives; previous whole-brain radiation; and brain-implanted devices such as DBS leads or aneurysm clips. The schedule of events and assessments is shown in Table 1. Demographic information, illness course, and medication history are shown in Table 2, Table 3, and Table 4 for the three subjects who completed the intervention phase of the protocol.

**Table 1:** Treatment protocol

	Screen	Baseline	Week			Month		
			1	2	3	6	9	12
Clinical scales	X	X	X	X	X	X	X	X
Cognitive tests	X				X			X
MRI	X		X		X	X	X	X
PET/CT	X							X
Responsive neurostimulation treatment		X						

**Table 2:** Subject demographics and characteristics ADD, attention deficit disorder; BP, bipolar disorder; D, divorced; ECT, electroconvulsive therapy; ETOH, alcohol; MDD, major depressive disorder; PD, personality disorder; S, single

Demographics	Subject 1	Subject 2	Subject 3
Age at the time of study entry	60	35	65
Sex/race	M/White	F/Asian	M/White
Marital status	D	S	S
Age first received any psychiatric care	37	12	20
Diagnoses prior to BP diagnosis	MDD and conduct disorder	MDD and avoidant PD social anxiety disorder	Schizoid PD, ADD, MDD, and dysthymia
Substance abuse (last used)	Marijuana (6 months prior to study)	None	ETOH/cocaine (10 years)
Family history			
Bipolar disorder	None	Two suspected: mother and maternal aunt	One suspected: maternal aunt
Other familial Axis 1 diagnoses	MDD in the mother, sister, and maternal uncle	Substance abuse by a maternal cousin	Postpartum depression in the maternal aunt and MDD in the maternal niece
Completed suicide	One maternal uncle	One maternal cousin	None
ECT	None	None	Maternal aunt

**Table 3:** Illness course BP, bipolar disorder; CGI-S, Clinical Global Impression-Severity; GAF, Global Assessment of Functioning; MDE, major depressive episode; N/A, not applicable; STEP-BD, Systematic Treatment Enhancement Program for Bipolar Disorder

Subject	1	2	3
Followed in the STEP-BD program since	2003	2002	N/A
Age at onset	Mid-30s	16	15
Age of diagnosis (BP1 or BP2)	45	24	54
Age of the first hypomanic or manic episode	42	16	20
Date of onset for the current MDE	2005 (observed)	2006 (observed)	1997 (reported)
Longest period of observed improvement: (CGI-S scores ≤2 and GAF ≥60)	10 months, 2004-2005	5 months, 2006	N/A
Number of prior MDEs	3 (chronic)	>25	>5
History of a psychotic mood episode	With mania	None	With MDE
History of rapid cycling	-	+	+
Last manic or hypomanic episode	2004	2004	2009
Number of hypomanic or manic episodes	3 manic episodes	>5	>3
Hypomania and/or mania during the study	1 manic episode	None	None
Number of past suicide attempts	2	3	None known
Number of past hospitalizations	3	>5	4

**Table 4:** Treatment history * The first acute/continuation ECT course was discontinued after 14 total treatments due to poor response and difficult-to-control paroxysmal atrial fibrillation with the last two ECT treatments. Two other acute courses were attempted in 2007 and 2009 but were discontinued after the first treatment due to the return of atrial fibrillation. ARP, aripiprazole; BPN, bupropion; BRF, brief; CBT, cognitive behavioral therapy; CBZ, carbamazepine; CTP, citalopram; DBT, dialectical behavior therapy; DLPFC, dorsolateral prefrontal cortex; DLX, duloxetine; eCTP, escitalopram; FLX, fluoxetine; LT, long term; LTG, lamotrigine; MDF, modafinil; MRT, mirtazapine; MT, motor threshold; NFD, nefazodone; OFC, olanzapine/fluoxetine combination; OLZ, olanzapine; PD, psychodynamic; PRX, paroxetine; QTP, quetiapine; rMDF, armodafinil; RSP, risperidone; SRT, sertraline; ST, supportive; TCA, tricyclic antidepressant; TCN, tranylcypromine; TMS, transcranial magnetic stimulation; tSLG, transdermal selegiline; tx, treatments; VEN, venlafaxine; VPA, valproate; ZPN, ziprasidone

Subject		1	2	3
Treatment at study entry	Mood stabilizer	OLZ (OFC) (6 mg)	ZPN (10 mg) and clozapine (37.5 mg)	Lithium (300 mg)
	Antidepressant	FLX (OFC) (50 mg), BPN (150 mg), and SRT (100 mg)	None	None
At the end of the study	Mood stabilizer	OLZ (OFC)	ZPN and clozapine	Lithium
	Antidepressant	FLX (OFC)	None	None
	ECT	0	8	0
Lifetime	Mood stabilizers	Lithium, VPA, LTG, OLZ, ARP, ZPN, and RSP	Lithium, VPA, CBZ, LTG, OLZ, ARP, ZPN, QTP, and RSP	Lithium, LTG, VPA, QTP, RSP, and intolerance ARP
	Antidepressants	TCN, tSLG, FLX, SRT, BPN, DLX, VEN, and MDF	PRX, VEN, FLX, CTP, MRT, BPN, and rMDF	eCTP, CTP, FLX, BPN, NFD, DLX, and >1 TCA
	Psychotherapy	LT: ST and PD; BRF: CBT and DBT	LT: ST and CBT; BRF: CBT and DBT	LT: ST and PD
	Prior ECT courses	1	1-2 years maintenance	3*
	Total number of ECT tx prior to study	14	56	None known
	Total number of ECT treatments during the study	0	8	0
	TMS 120% MT 10 Hz left DLPFC	0	0	One course of 25 treatments

All patients were interviewed using the Mini International Neuropsychiatric Interview (MINI) Structured Clinical Interview for DSM (SCID) to confirm the DSM-IV diagnosis of bipolar disorder in the depressive phase. Clinical rating scales used were the 17, 21, and 24-item HDRS (range 0-52), the Clinical Global Impression-Severity (CGI-S, range 1-7) and Clinical Global Impression-Improvement (CGI-I, range 1-7), and the Young Mania Rating Scale (YMRS, range 0-60). These clinical assessments were done at weeks 1, 2, and four weeks, and two, three, six, nine, and 12 months post-SRS. At baseline prior to the SRS procedure, patients completed a neuropsychiatric battery using the Delis-Kaplan Executive Function System (DKEFS), the Wechsler Adult Intelligence Scale III (WAIS-III) digit span, and the California Verbal Learning Test II (CVLT-II). These cognitive tests were repeated at the three- and 12-month follow-up visits. Adverse events were reviewed and logged with each visit.

SRS planning

A preprocedure CT of the head without contrast was obtained, as were T1-weighted, T2, and fluid-attenuated inversion recovery (FLAIR) sequence MRI data sets. The target location within the SGC was anatomically determined using a digital fusion of the CT data set with the T1-weighted MRI data set, and the accuracy of the fusion was verified by a neurosurgeon supervising the treatment. The target regions were then digitally demarcated by representing them as two targeted spheres (left and right), each 5 mm in diameter and 66 mm^3^ in volume. The 60 Gy was prescribed to the target volumes in the 80% isodose line by an automated beam planning process that used approximately 100 beams for each target; therefore, the maximum dose was 75 Gy.

SRS treatment

Radiation was administered to the two bilateral SGC targets on two consecutive days. During each procedure, the patient was supine on the treatment table with their head immobilized within a custom-fitted face mask. The patient was then registered within the stereotactic space using image-based registration. Once proper registration was confirmed, radiation delivery began in accordance with the treatment plan. Following each treatment, the patient was given 4 mg of dexamethasone to minimize the likelihood of potential nausea or edema associated with the treatment. The patient was then discharged home with follow-up instructions. A brief physical exam was conducted at the first follow-up appointment one week after the procedure by a neurosurgeon.

Imaging

Follow-up MRIs, which included T1, T2, and FLAIR sequences, were done at one week, three, six, nine, and 12 months in order to identify structural brain changes.

## Results

Subjects had a mean decrease in HDRS-17 of 27% between baseline and one-year post-intervention follow-up.

**Figure 1:**
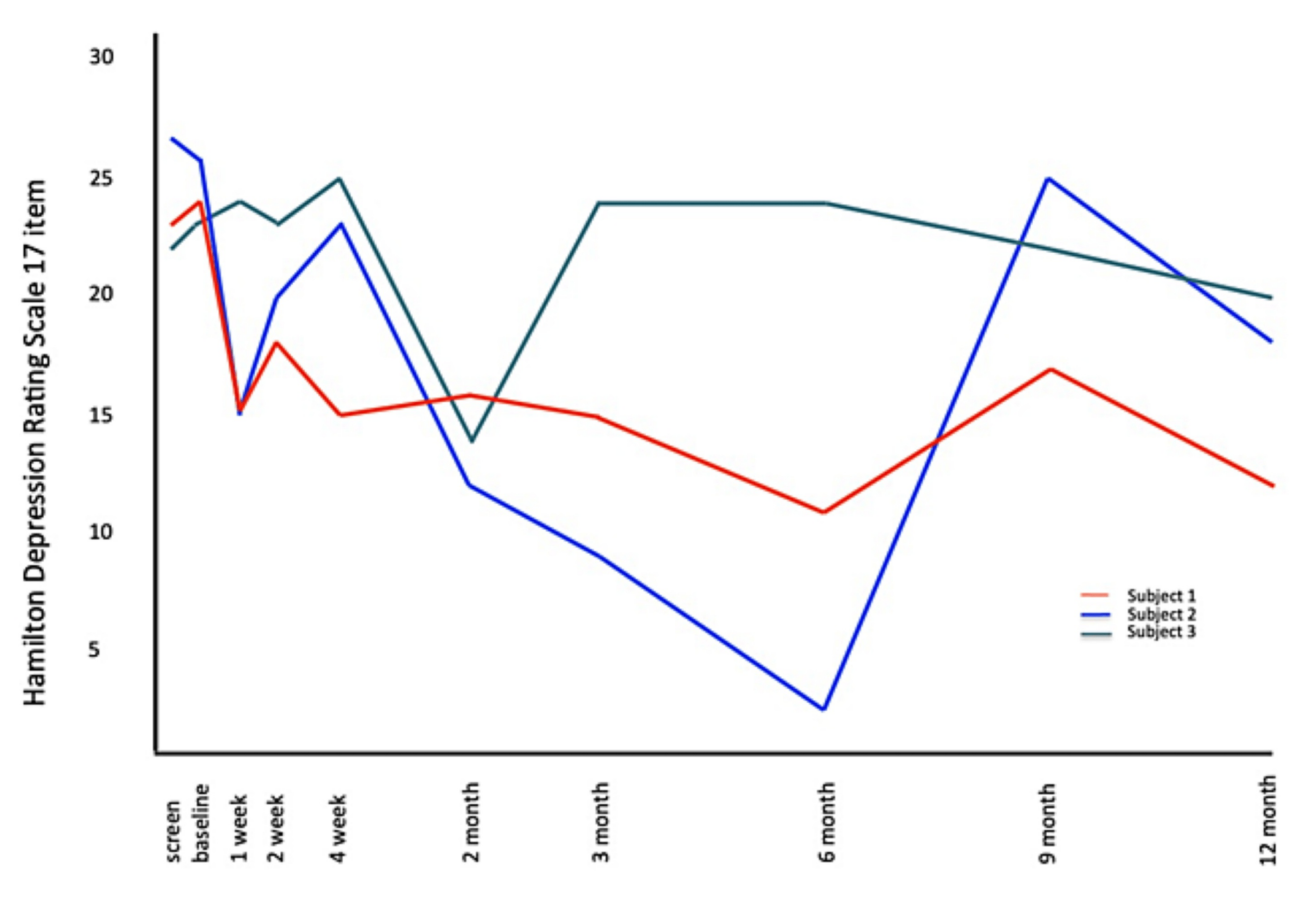
HDRS-17 scores from screening through 12 months post-SRS HDRS, Hamilton Depression Rating Scale; SRS, stereotactic radiosurgery

**Figure 2:**
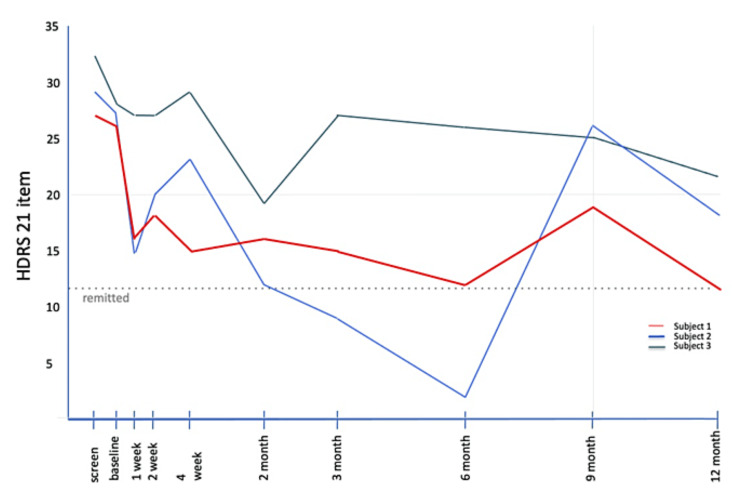
HDRS-21 scores from screening through 12 months post-SRS HDRS, Hamilton Depression Rating Scale; SRS, stereotactic radiosurgery

**Figure 3:**
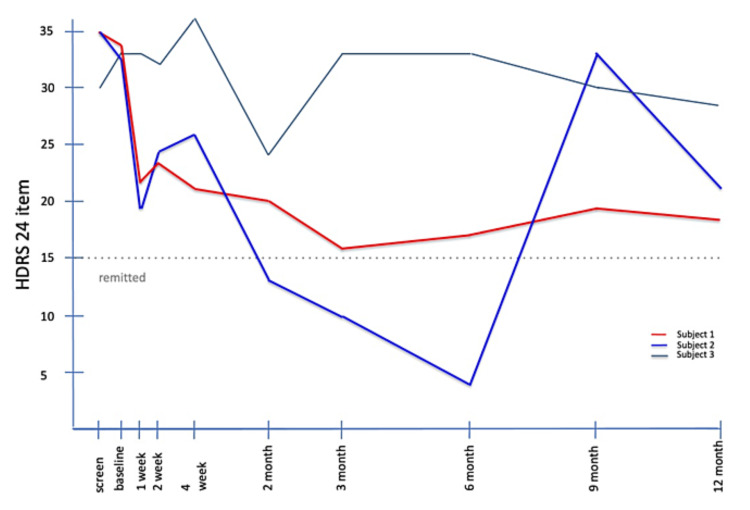
HDRS-24 scores from screening through 12 months post-SRS HDRS, Hamilton Depression Rating Scale; SRS, stereotactic radiosurgery

Subject 1 met response criteria (≥50% reduction) on the HDRS-17 and HDRS-21 at six (54%) and 12 months (50%) post-intervention. He met response criteria on the HDRS-24 at three-, six-, and 12-months post-SRS. Subject 2 met response criteria at the two and three-month assessments and was remitted at six months on all HDRS scales. Subject 3 showed a pattern of decreasing severity of symptoms up to the three-month visit, but this was not sustained, with a return to baseline severity at 12 months. HDRS-17, HDRS-21, and HDRS-24 scores are found in Figure 1, Figure 2, and Figure 3.

**Figure 4:**
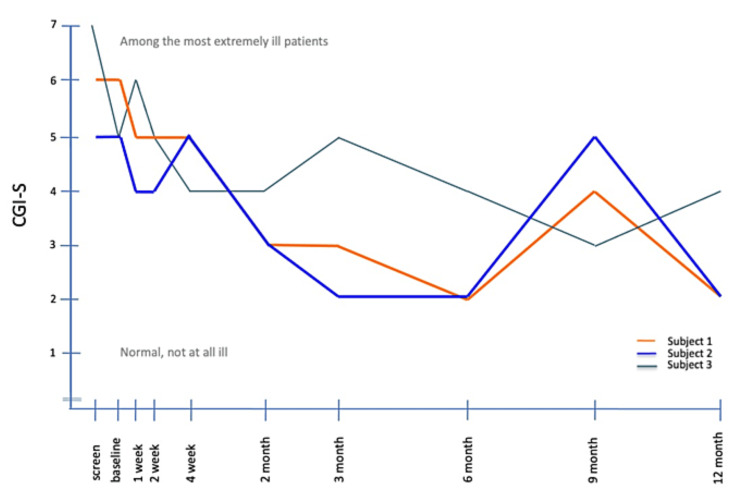
CGI-S scores from screening to the end of the study assessment CGI-S, Clinical Global Impression-Severity

Both Subjects 1 and 2 showed improvement on the CGI-S shown in Figure 4. Their scores decreased from 5 (severely depressed) to 2 (borderline mentally ill) at 12 months. Both subjects also showed marked improvement on the CGI-I, with both achieving a score of 1 (very much improved) at 12 months (not shown). Subject 1 had an elevation on the YMRS scale at six and nine months, corresponding to a switch into hypomania and then mania shortly after he self-discontinued the mood stabilizer olanzapine/fluoxetine combination. Although Subject 3 did not show an improvement on the depression scales, he did improve on both the CGI-S and CGI-I.

**Figure 5:**
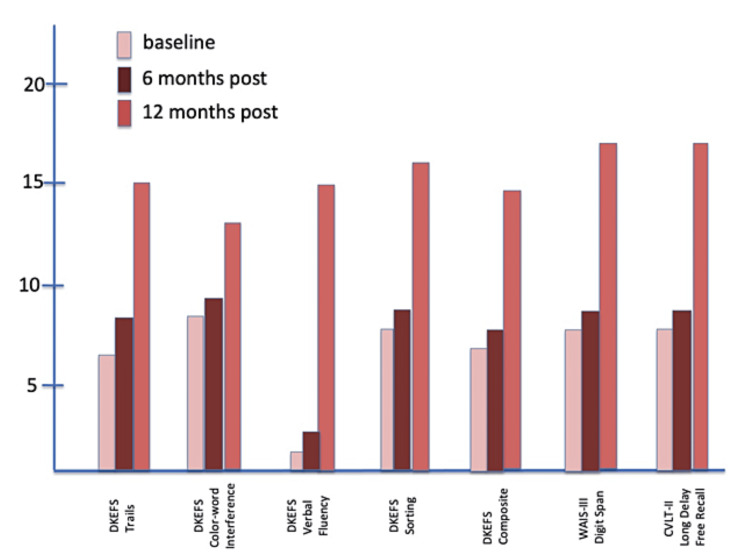
Composite DKEFS CVLT-II scores at baseline, six months, and 12 months for Subject 1 CVLT-II, California Verbal Learning Test II; DKEFS, Delis-Kaplan Executive Function System; WAIS-III, Wechsler Adult Intelligence Scale III

**Figure 6:**
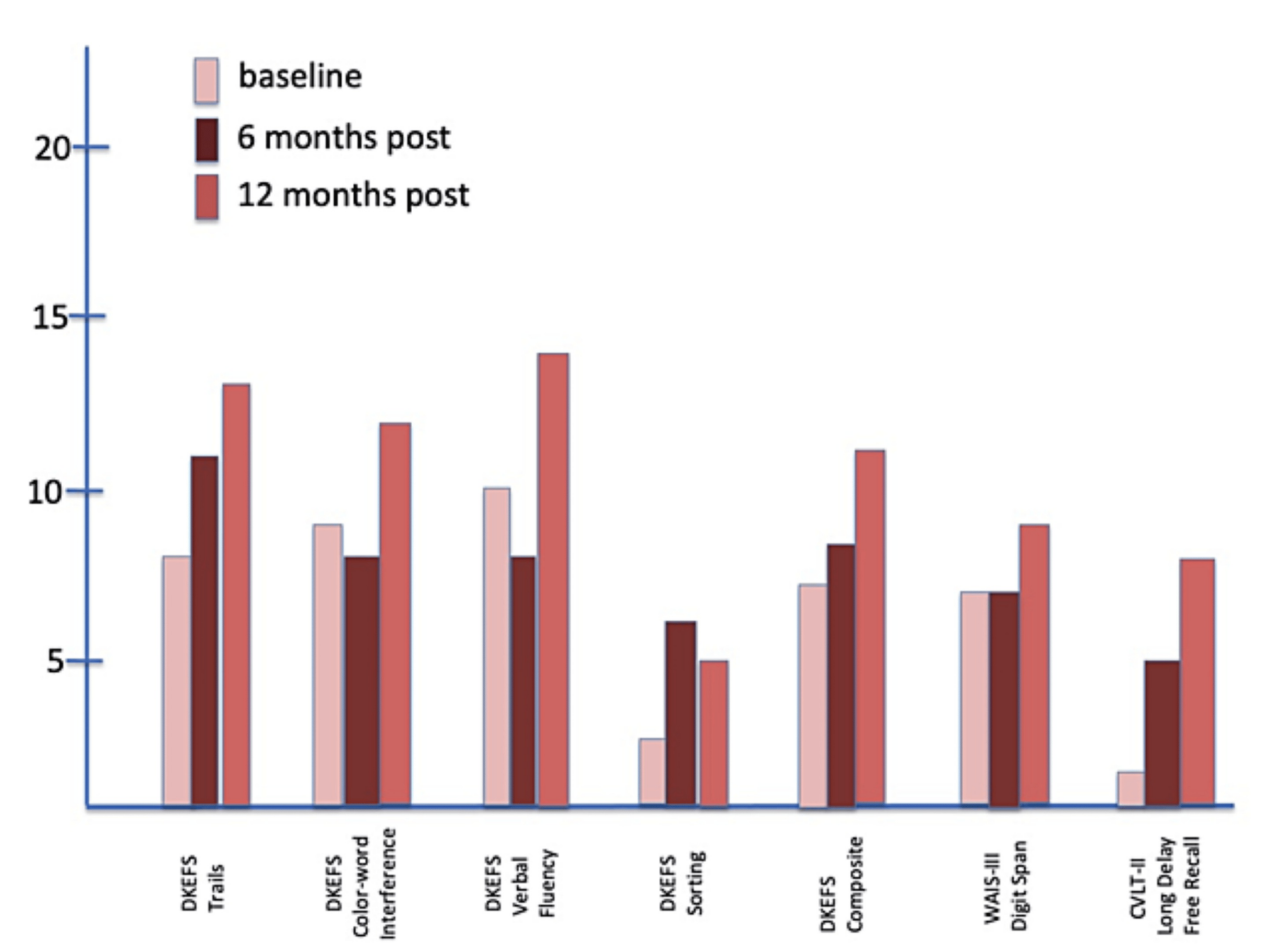
Composite DKEFS CVLT-II scores at baseline, six months, and 12 months for Subject 2 CVLT-II, California Verbal Learning Test II; DKEFS, Delis-Kaplan Executive Function System; WAIS-III, Wechsler Adult Intelligence Scale III

**Figure 7:**
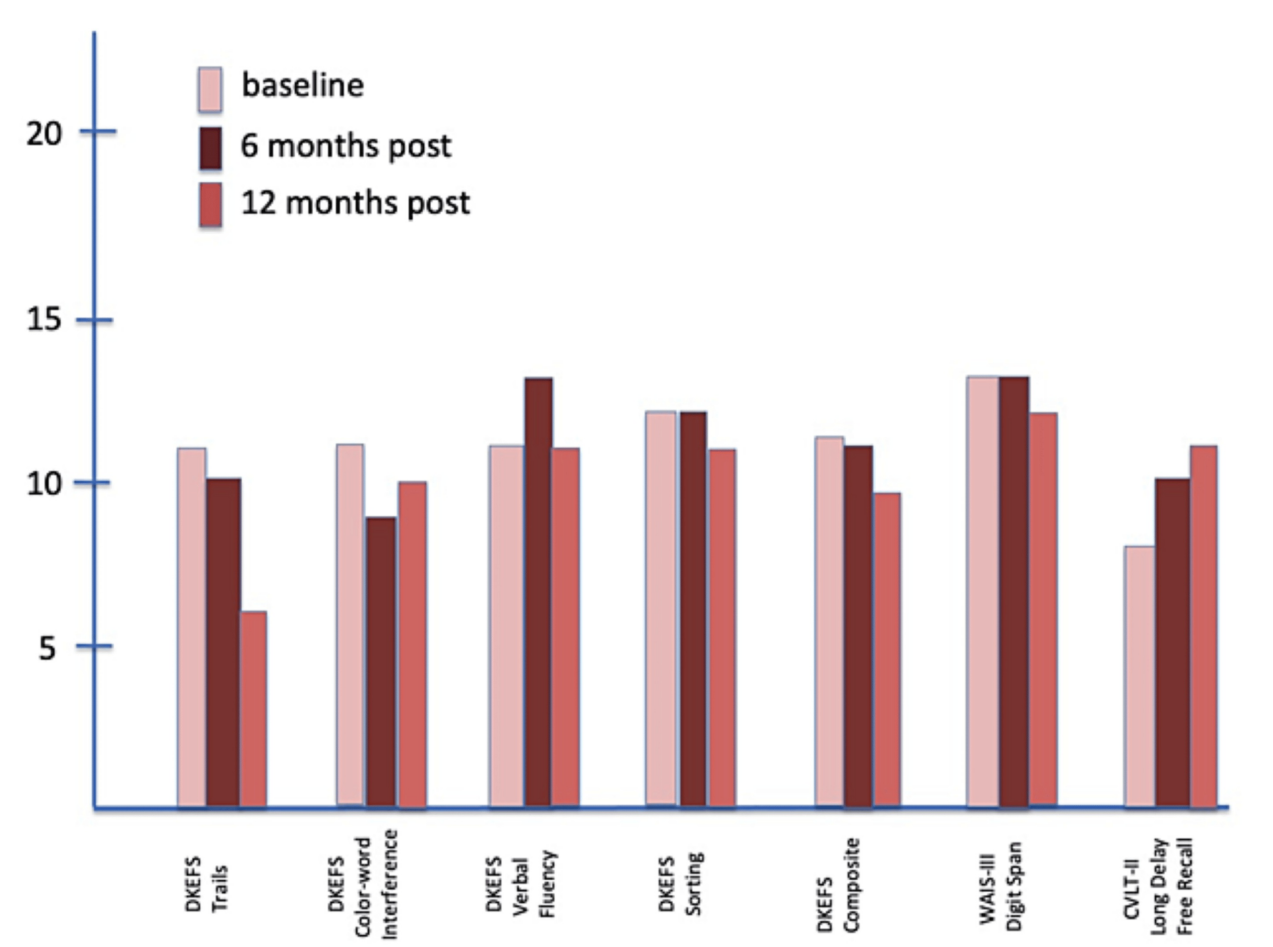
Composite DKEFS CVLT-II scores at baseline, six months, and 12 months for Subject 3 CVLT-II, California Verbal Learning Test II; DKEFS, Delis-Kaplan Executive Function System; WAIS-III, Wechsler Adult Intelligence Scale III

Neurocognitive outcomes are shown in Figure 5, Figure 6, and Figure 7. The DKEFS scores are composite scores for each type of assessment battery: trails, color-word interference, verbal fluency, sorting, and an overall composite score. The WAIS-III digit span and CVLT-II long delay-free recall outcomes follow the DKEFS scores. Serial testing at baseline, six, and 12 months showed improvement from baseline to the end of the study for Subject 1. Neither Subject 2 nor Subject 3 showed improvement or a decrease in performance on any cognitive domain tested.

Clinical outcomes for both Subjects 1 and 2 over the 12-month follow-up period were complicated in each case by a circumstance that directly and negatively affected a consistent pattern of improvement seen over the first six months. Just after the six-month assessment, Subject 1 self-discontinued the olanzapine/fluoxetine combination (OFC) due to financial concerns. Four weeks later, he became hypomanic, and despite starting valproate at that point, within six weeks, he was hospitalized with a full manic episode. He was eventually restarted on OFC, along with lithium and lamotrigine. Shortly after restarting OFC, there was again a marked improvement in clinical assessments that continued until the 12-month time point.

Just before the two-month assessments post-SRS, Subject 2 experienced a family death (suicide). This loss had particular meaning for the subject, as she and this family member had made a pact with each other not to commit suicide. The subject’s resilience in the context of such a significant loss was supported by her own observation that she was grieving but did not feel “depressed” after the suicide. She did rate higher on the HDRS and CGI-S and lower on the CGI-I at the two-month assessment, but then regained earlier improvement by the next visit.

Near the nine-month time point, Subject 2 was hospitalized following decompensation due to another discrete and severely stressful precipitant. She received a course of ECT treatment (four acute and four at weekly intervals) and subsequently showed improvement; however, she did not achieve the level of sustained improvement she had experienced earlier in the trial.

Both Subjects 1 and 2 were part of the Systematic Treatment Enhancement Program for Bipolar Disorder (STEP-BD) study at Stanford. CGI-S scores (not shown) were available in the medical record for each clinic visit from 2003 for Subject 1 to 2002 for Subject 2. Subject 1 had a pattern of increasing severity and disability over the approximately eight years prior to SRS. Subject 2 had an illness course characterized by wide fluctuations in depressive severity and only five brief periods of improvement over the first four years of follow-up. Subject 2 then had a 33-month course of acute ECT followed by maintenance ECT (119 treatments total) prior to entering the study. Following the SRS procedure and during the study, the patient had a brief course of ECT following the stressful precipitant mentioned above. She has not required ECT over the nearly three years post-SRS.

Overall, all three subjects reported a subjective improvement after the SRS procedure. Subjects 1 and 2 have since requested retreatment. Subject 3 recalled having improved by the end of the study period (which is more consistent with the CGI scores than depression scales). He has since responded to acute and maintenance low-frequency TMS (1 Hz, 1600 pulses per session, over the right dorsolateral prefrontal cortex) over the last 13 months (88 treatments in total).

**Figure 8:**
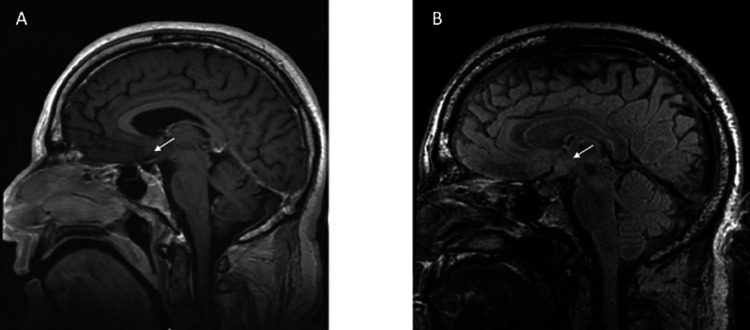
Subject 1 MRI brain at (A) 12 and (B) 27 months post-SRS treatment The white arrows show the area of lesioning in the subgenual cingulate. SRS, stereotactic radiosurgery

**Figure 9:**
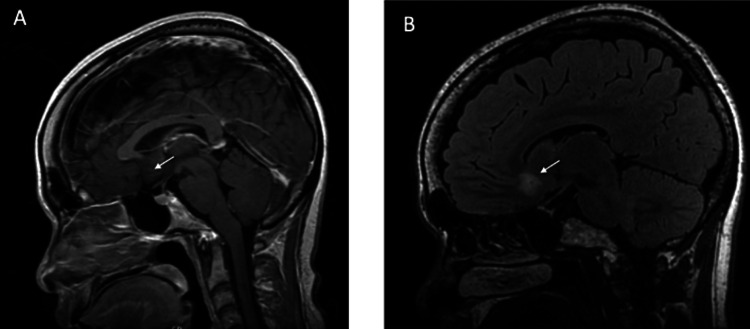
Subject 2 MRI brain at (A) 12 and (B) 32 months post-SRS The white arrows show the area of lesioning in the subgenual cingulate. SRS, stereotactic radiosurgery

**Figure 10:**
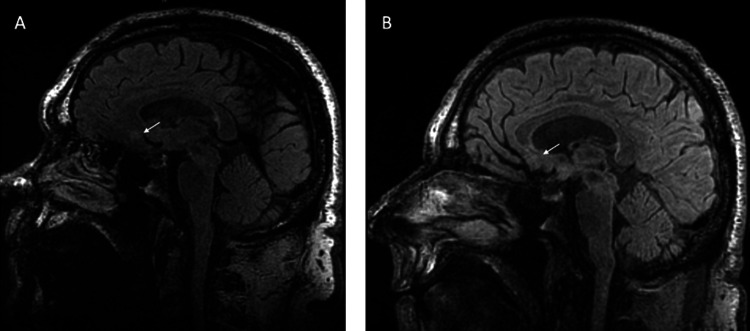
Subject 3 MRI brain at (A) six and (B) 21 months post-SRS The white arrows show the area of lesioning in the subgenual cingulate. SRS, stereotactic radiosurgery

MRI studies are shown in Figure 8, Figure 9, and Figure 10. In Subject 1 (Figure 8), no abnormalities were visible up to 38 months post-SRS. In Subject 2, a signal abnormality on the FLAIR sequence MRI consistent with edema was observed at 32 months post-SRS; however, he remained neurologically intact. Subject 3 also showed a signal abnormality on the FLAIR sequence MRI consistent with edema at nine and 12 months post-SRS; however, at 22 months post-SRS, the majority of the edema had resolved. Despite these observed signal abnormalities, the subjects did not suffer any adverse effects.

## Discussion

In this paper, we present the results of a safety and feasibility study using SRS targeting SGC in patients with TRBD. These preliminary data suggest that SRS is a safe and effective procedure for the treatment of TRBD. There was an apparent clinical benefit in depression severity in two subjects following the SRS procedure that persisted over the 12-month study. Performance on serial neurocognitive tests and in the overall functional capacity of all three subjects did not reveal any negative effects of treatment. We documented mood improvement during the study period that occurred in the absence of lasting lesions on MRI or adverse effects. Given that mood improvement was modest and transient, we do not advocate treating additional patients with the same parameters. Local inflammation visible on FLAIR (not suggestive of necrosis) in one of three patients at 32 months post-SRS was the only durable change visible at the last follow-up.

These changes, seen at nine and 12 months, resolved to a punctate lesion 22 months after SRS. The appearance of MR signal abnormalities was not associated with a change in his depressive symptoms or performance on the 12-month cognitive battery, nor was there any change observed that would reflect new impairments in judgment or insight.

Of interest, Subject 3 had a course of high-frequency TMS prior to study entry without benefit. Two weeks after the 12-month SRS study was complete, he began a course of right-side low-frequency TMS treatments (1 Hz, 10 minutes, 600 pulses per session, 120% motor threshold) and completely remitted over five weeks. He sustained improvement over the last 15 months with maintenance treatment.

Of the two subjects who had longitudinal data from visits to the Stanford Bipolar Clinic, the CGI-S data clearly illustrated the chronicity, severity, and disability of their depression. The clinical response to ECT in both patients was limited and not sustained. In the context of this long-term historical data, the improvement in HDRS and CGI scores post-SRS appeared to show a clinical benefit of the procedure when contrasted with the overall trend in clinical status seen prior to the study. The clinical response to SRS, both in terms of severity of depression and duration of improvement, appears to be more robust than that previously seen with ECT treatment for both subjects.

The apparent lesion seen in the SGC in Subject 3 at nine and 12 months and Subject 2 at a 32-month follow-up scan was unexpected. It may be that the SGC was more vulnerable to radiation injury, perhaps related to its hypermetabolism during the depressed state, progressive atrophy, and a history of chronic cocaine and alcohol use in both subjects. Due to its neuroprotective and anti-apoptotic effects in bipolar disorder as well as neurodegenerative disorders [34], pretreatment with lithium may reduce the likelihood of a lesion arising in the targeted area.

Histologically lesion-free changes accompanied by bidirectional neuromodulation are seen in large animal models with PET [36] and with depth electrodes and visual evoked potential [37]. If target upregulation is a viable therapeutic strategy, it may be that treating at doses of 10-30 Gy, for example, may yield more favorable results.

It is possible that a lesion in the SGC may have a therapeutic outcome. Lesion-based neurosurgery is known to be effective; improvement in mood occurred before the destructive lesion was discovered [38]. Recent animal work shows evidence of nondestructive functional upregulation of focal gray matter at doses below 40 Gy and dose-dependent partial lesioning above that level [39]. Neurosurgical procedures such as anterior cingulotomy, subcaudate tractotomy, and stereotactic limbic leucotomy have been used to successfully treat refractory bipolar disorder and depression, as well as obsessive-compulsive disorder [39]. A study of healthy volunteers showed that connectivity reconstructions from diffusion tensor imaging tractography data suggested that the shared connectivity between these surgical approaches was through the subgenual region [40]. Such studies may provide some insight into how targeted radiation to the SGC may have therapeutic value for the most difficult-to-treat patients with bipolar disorder.

Limitations

The major limitation of this study is that only three patients were enrolled, with limited follow-up. Given the limited sample size without a control group, it is difficult to say definitively that SRS for TRBD is both safe and effective. However, it is hoped that the positive results presented here will inspire a future trial with a larger number of patients.

The positive results presented here should inspire future clinical and basic science investigations. In the realm of basic science, more molecular studies are needed to better understand the impact of focused radiation on neural tissues. On the clinical side, trials with a larger number of patients and sufficient controls are needed to adequately show the safety and effectiveness of SRS in the SGC for the treatment of TRBD.

## Conclusions

These data from three subjects support the hypothesis that the SRS procedure does not seem detrimental and may provide some benefit for the treatment of TRBD. The radiation-related injury to SGC in Subjects 2 and 3 did not result in a worsening of depression or cognitive impairment. It is possible that a lower dose should be used in future trials. Overall, the procedure appears to have resulted in sustained clinical benefit in two subjects that exceeded the clinical outcomes seen with prior trials of medication and ECT for both subjects. This may be an indication that SRS could be a precisely targeted, noninvasive approach to the TRBD. More research is needed to further determine the safety and clinical efficacy of SRS for the treatment of this disease.
