# Correction: Treatment of Refractory Bipolar Depression With Stereotactic Radiosurgery Targeting the Subgenual Cingulate Cortex

**DOI:** 10.7759/cureus.c282

**Published:** 2025-09-04

**Authors:** Hugh B Solvason, Neelan J Marianayagam, Scott G Soltys, Alan F. Schatzberg, Charles DeBattista, Terence Ketter, Po Wang, Steven D. Chang, David Spiegel, John R Adler

**Affiliations:** 1 Department of Psychiatry, Stanford University School of Medicine, Stanford, USA; 2 Department of Neurosurgery, Stanford University School of Medicine, Stanford, USA; 3 Department of Radiation Oncology, Stanford University School of Medicine, Stanford, USA

This article's ethics statement has been corrected to include the approval number (10186), which was erroneously omitted from the initial submission.

